# Clinical Performance of Template-assisted Monochromatic Structural Colored Versus Universal Multishade Direct Composite Veneers Over 3 Years: A Randomized Clinical Trial

**DOI:** 10.4317/jced.63075

**Published:** 2025-09-01

**Authors:** Ali A Elkaffas, Abdullah Alshehri, Abdullah Ali Alqahtani, Shahad Saleh Alghannam, Hamad Algamaiah, Abdulrahman Alshabib, Laila Taher Kashkosh, Rania Bayoumi, Saleh Alhindi, Mohammed Abd El-Ghany Mohammed

**Affiliations:** 1 Dept. of Conservative Dental Sciences, College of Dentistry, Prince Sattam Bin Abdulaziz University, Alkharj, Saudi Arabia; 2 Dept. of Operative Dentistry, Faculty of Dentistry, Mansoura University, Mansoura, Egypt; 3 Dental Hospital, College of Dentistry, Prince Sattam Bin Abdulaziz University, Alkharj, Saudi Arabia; 4 Department of Restorative Dental Science, College of Dentistry, King Saud University, Riyadh, Saudi Arabia; 5 Dept. of Conservative Dental Sciences, College of Dentistry, Tanta University, Tanta, Egypt; 6 Biomaterials Department, Faculty of Dental Medicine for Girls, Al-Azhar University, Cairo, Egypt; 7 Assistant Professor, Dept. of Conservative Dental Sciences, College of Dentistry, Tanta University, Tanta, Egypt

## Abstract

**Background:**

We conducted a clinical trial using a modified split-mouth, double-blind, randomized approach to clinically assess template-assisted monochromatic structural colored versus universal multishade direct composite veneers over a 36-month period.

Materials and Methods: A total of 88 direct composite veneers from 20 participants were included per the inclusion criteria. Each patient received at least one pair of the two types of direct veneers in two equal groups (n = 44): (group I): monochromatic structural colored veneer (OMNICHROMA) with palfique adhesive and (group II): universal multishade veneer (Ceram.x spectra) with Prime & Bond adhesive using U-veneer templates. Window-type veneer preparations were performed on the labial surface of anterior teeth (depth: 0.3–0.5 mm). Clinical assessment was conducted per modified United States Public Health Service (USPHS) criteria. The marginal integrity criterion was evaluated by scanning electron microscopy of an inverse replica of 32 randomly selected veneer restorations.

**Results:**

The Friedman and Wilcoxon signed-rank tests revealed significant differences between group I and group II in marginal adaptation, marginal discoloration, surface texture, and color match (*p* <0.05). However, there no significant differences in fracture type or anatomical form existed between the two groups (*p* >0.05). The Mann‒Whitney U test indicated no significant differences between the groups across USPHS criteria (*p* >0.05). No secondary caries or hypersensitivity cases were reported during any evaluation period. The unpaired t test revealed no significant difference in the mean gap width between the two groups (*p* = 0.218 and 0.236, respectively). Spearman’s correlation test, conducted on the related criteria in groups I and II after 12, 18, and 36 months of follow-up, revealed a positive relationship between the evaluated criteria.

**Conclusions:**

Monochromatic structurally colored and universal multishade direct composite resin veneers demonstrated comparable satisfactory clinical performance by the end of the study period.

** Key words:**Composite, Monochromatic, Multishade, Veneers, USPHS criteria.

## Introduction

In recent decades, increasing demands have increased the use of direct resin composites in aesthetic restorative dentistry [1]. Recent advancements in adhesive dentistry have led to the development of materials and procedures designed to restore the natural appearance of teeth, particularly in the anterior region. The use of more aesthetically pleasing materials that mimic natural tooth structures enables clinicians to craft restorations that closely resemble natural dental tissues [2].

Several dental issues, including carious lesions, tooth discoloration, tooth fractures, and misaligned teeth, can severely impair smile harmony and aesthetic appeal, which in turn affects quality of life [3]. Direct composite resin veneers can be an appealing option for enhancing the aesthetic appearance of damaged teeth, as they allow the operator to monitor and assess the entire restoration procedure, from selecting the shade to shaping the final morphology [4].

Direct resin veneering with composites involves applying resin directly onto prepared tooth surfaces, followed by artistic sculpting to address aesthetic issues related to color, anatomy, and morphology [5]. Direct composite veneering offers numerous benefits: it is a chair-side procedure that eliminates the need for multiple visits or luting agents, is minimally invasive, preserves natural tooth structure, allows for easy repairs, and is cost-effective since it does not incur laboratory expenses. Furthermore, the abrasion rates of direct composite veneers are comparable to those of natural tooth structures [6].

Aesthetic considerations primarily involve precise color matching and adequate adaptation between the restoration and adjacent hard dental tissues. Moreover, surface texture, restoration location, and contour are other critical factors for achieving favorable outcomes. Since natural teeth display color variations, manufacturers have developed composite resins with various shades, often referencing the Vita Classical shade guide [7]. Additionally, resin composites are available in a range of opacities and are typically categorized as enamel, translucent, body, or opaque, depending on the dentin. These materials replicate the optical properties of enamel and dentin and are recommended for different tooth regions [8].

The layering approach for direct resin composite restorations has been recommended since 1980. In this procedure, composites with varying chromas and opacities are used for each layer to replicate the optical characteristics of a natural tooth [9]. Although the layering technique has been shown to produce satisfactory results for color matching, this method is considerably more complex than a standard procedure that utilizes one or two shades. It requires a longer chair-side time and advanced restorative skills [10].

Clinicians are seeking composite materials and restorative techniques that enable the execution of streamlined clinical protocols to reduce technique sensitivity, minimize chair time, and enhance the aesthetic quality of the final results [11]. Owing to the complex nature of color selection and its dependence on operator and environmental variables, there has been a movement to standardize shade selection through the development of so-called universal composites. Given their limited Vita shade options and uniform opacity, developers recommend applying these composites in a single shade increment to match various tooth hues [12].

The novel single-shade resin restorative composite OMNICHROMA, which mimics the appearance of a natural tooth structure and features 260 nm spherical fillers, has recently been developed via smart chromatic technology [13]. Its wide color matching capability eliminates the need for a shade selection procedure, reducing the inventory of composite resins and allowing clinicians to minimize chair time, reduce the waste of unutilized resin composite shades, and minimize their reliance on shade selection procedures [14].

Although *in vitro* screening has been conducted, clinical testing of restorative materials is fundamental for ascertaining their durability [15]. Even *in vitro* investigations with carefully crafted simulations of clinical scenarios differ markedly from *in vivo* situations. Many interactive clinical variables in the oral cavity related to the oral environment and tooth substrate cannot be simulated *in vitro* [16]. This study’s null hypothesis is that there is a significant difference in the clinical performance between monochromatic structurally colored and universal multishade direct composite veneers.

## Material and Methods

The materials used in this study are summarized in  Table 1, including their chemical composition, manufacturer information, and associated websites.

- Study design

**Figure 1:**
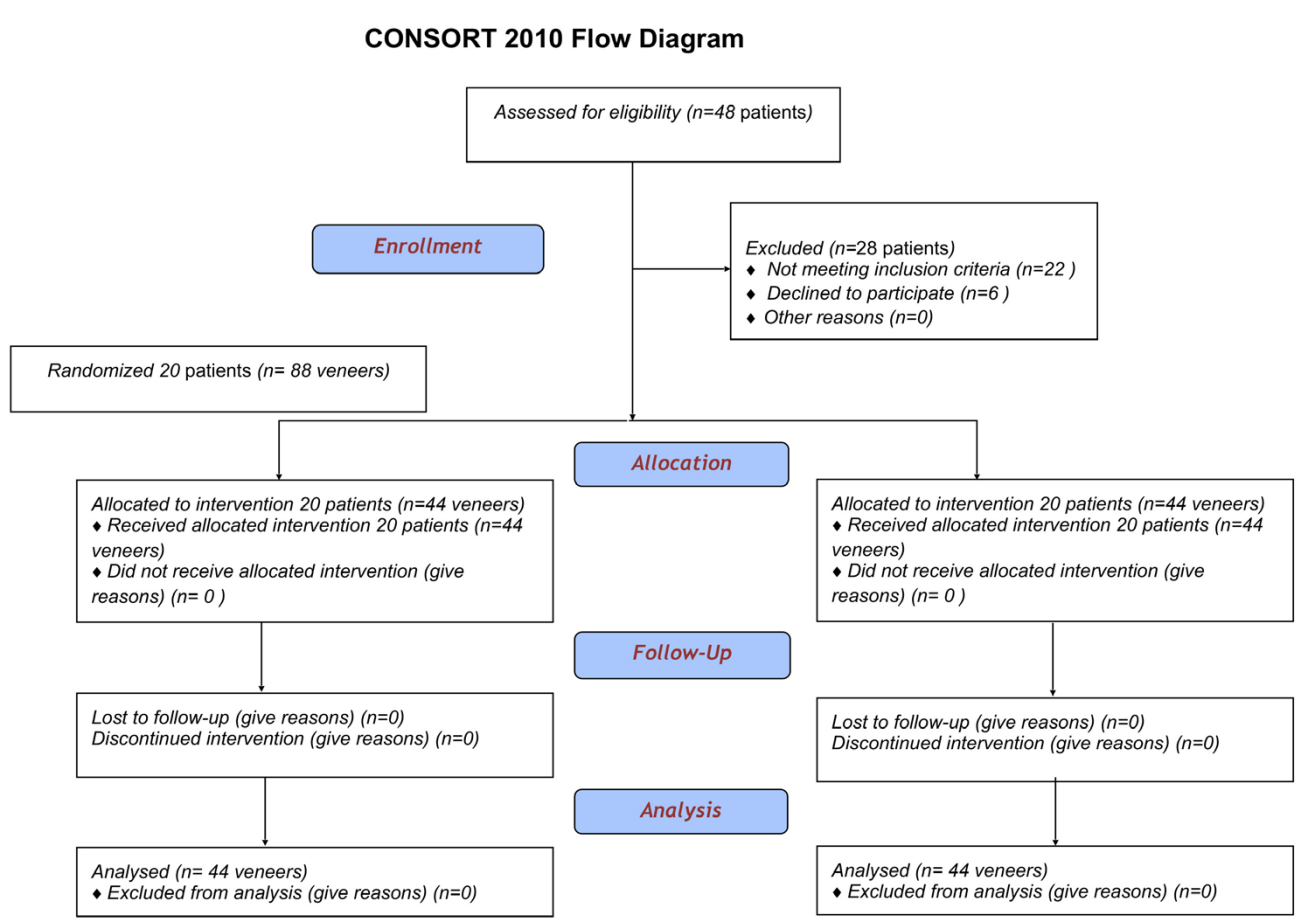
Diagram showing CONSORT flow chart.

This clinical trial was conducted using a double-blind, randomized, modified split-mouth design. This was a parallel-group study with an allocation ratio of 1:1. The study was designed in accordance with the Consolidated Standards of Reporting Trials (CONSORT) statement of 2010 (Fig. 1) [17].

- Study setting

This study was conducted at the clinic of the Department of Restorative Dentistry, Faculty of Dentistry, Tanta University.

- Sample size

The sample size was calculated via the G Power version 3.1.9.6 software statistical package, with a desired alpha of 0.05, a statistical power of 80%, an equivalency limit of 20%, and an effect size of 0.307. The study minimally required 32 veneer restorations in each group, distributed across 16 patients who had at least four discolored vital anterior teeth (for a total of 64 veneers in both groups). The sample size was increased to 44 veneers for each group, which were distributed among 20 patients, resulting in a total sample size of 88 veneers. Sixteen patients had 64 upper incisor veneers placed, whereas four patients had 24 upper incisor and canine veneers placed. This oversizing aimed to compensate for potential failures in follow-up visits and to enhance the validity of the results.

- Ethical considerations

All the steps of the treatment procedures, benefits, drawbacks, expectations, and assumed complications were explained to selected patients. Informed consent was obtained from all participants per the guidelines on human research adopted by the Research Ethics Committee, Faculty of Dentistry, Tanta University. The protocol for this clinical research was approved by the committee after fulfilling the necessary requirements, with code #R-RD-3-25-3195. All procedures performed in this study were conducted in accordance with the tenets of the Declaration of Helsinki.

- Patient selection

A comprehensive list of inclusion and exclusion criteria was employed, resulting in the inclusion of 20 volunteers of both sexes (average age: 25–40 years) in the current study. These participants were selected from the clinic of the Restorative Dentistry Department at Tanta University’s Faculty of Dentistry. They received oral hygiene instructions before restorative treatment; when necessary, they were referred to the periodontology department for scaling and polishing.

• Inclusion criteria

− Proper oral hygiene.

− Essential anterior teeth stained due to fluorosis, minor tetracycline discoloration, hypoplasia, or cracks.

− Proper periodontal health.

− Availability for follow-up periods.

• Exclusion criteria

− Patients using drugs that can alter tooth color.

− Significant discoloration of teeth.

− Parafunctional habits such as tooth clenching or grinding.

− Endodontically treated or nonvital teeth.

- Randomization and allocation concealment

A randomization list was prepared (www.randamization.com). Each patient was assigned an identifying code, and two composite veneer options (OMNICHROMA and Ceram.x spectra) were selected randomly from a list where each participant received at least one pair of the two types of direct veneers (modified split-mouth technique). A blocked list was generated, and a randomization code was created according to the two veneer options [18]. Allocation concealment was achieved through the use of numbered cards stored in opaque, sealed envelopes. Aluminum foil was placed inside the envelopes to ensure that they were impervious to harsh light. Each patient received a separate sealed opaque envelope from a secretary who was not involved in the clinical procedures. The envelopes contained a link indicating the type of restoration used, along with the randomization code that determined the participant’s group on the day of the operation.

- Blinding

This clinical study employed a double-blind design, where patients and evaluators not involved in the restorative procedures were blinded to the tested groups; however, the operator was not blinded to these groups.

- Restorative procedures

Initially, the shade was visually chosen in the dental office using the Vita Classical shade guide (Lumin Vaccum, Vitapan, Vita Zahnfabrik, Germany). Later, a digital spectrophotometer (VITA Easyshade® V, Vita Zahnfabrik, Germany) was used, referencing the unaffected areas of the teeth, neighboring teeth, or opposing teeth [19]. The average shade determination mode of the VITA Easyshade® V calculates an average base tooth shade from multiple measurements across several areas of the tooth, providing a single value. This is important because variances in enamel thickness at various anatomical sites can affect final shade reproduction. Putty silicon was utilized to create an index for each patient using condensation silicon impression material (Silaxil putty, Sesto Fiorentino, Florence, Italy). Each index was cut vertically at the midpoint of the labial surface on each side to assess the preparation of the labial surfaces [20].

Window-type preparations for direct composite veneers were performed on the labial surface of anterior teeth to a depth of 0.3–0.5 mm, guided by depth cut marks (Depth cutter wheels, Komet, Germany). This approach utilized cutter depth wheels for minimal preparations of approximately 0.3 mm in the cervical third and 0.5 mm in the middle and incisal third, ensuring even preparation thickness while preserving the incisal edge [21]. A tapered diamond stone (Komet, Germany) with a 0.3 mm diameter was employed to remove the remaining islands of enamel until the depth of the orientation grooves was reached. Labial reduction was conducted in three distinct planes, namely, the cervical, middle, and incisal planes, to follow the natural contour of the labial surface [22]. The operating field was completely isolated via a rubber dam (Sanctuary Dental dam, Malaysia).

A light chamfer finish line was created at the cervical area to ensure an adequate seal of the composite veneers. The veneer preparations extended just beyond the mesilabial and distolabial line angles and were made slightly labial to the contact point interproximally. All margins were positioned at the gingival margin to provide optimum esthetics and maintain good periodontal health. The internal line angles were slightly rounded. Each preparation was then verified vertically with the silicone index to check the amount of labial reduction [23].

The central incisors of each patient were treated with the same type of veneer material, either OMNICHROMA or Ceram.x spectra™, according to randomization and allocation concealment to prevent noticeable shade changes. Each patient received at least one pair of each type of direct veneer. Eighty-four direct veneers were applied to the upper incisors of 16 treated patients. Four patients received 24 direct veneers for their upper incisors and canines throughout the study. The distributions of the restorative materials and locations are listed in  Table 2. A total of 88 direct composite veneers were divided into two equal groups (n = 44) as follows: group (I): OMNICHROMA monochromatic with Palfique bond self-etching adhesive; group (II): Ceram.x spectra™ ST universal multishade with Prime&Bond universal self-etching adhesive. The prepared enamel surfaces were etched with 37% orthophosphoric acid gel (N-Etch, Ivoclar, Vivadent, Schaan, Liechtenstein) for 15 seconds, rinsed with a water stream for 20 seconds, and dried with jets of oil-free air spray. Teflon tape (PTFE-TAPE DIN-EN, Germany) was placed and wrapped around adjacent teeth during adhesive and restorative procedures to protect them.

For group I:

A layer of self-adhesive palfique bonds was applied to the prepared enamel surfaces, which were then brushed for ten seconds with a disposable microbrush. This was followed by gentle air dispersion for five seconds and light curing for ten seconds using a light emitting diode (LED) curing device (Woodpecker Dental LED D, Wireless LED Lamp Curing Light, China) with an output intensity of 850–1000 mW/cm2. OMNICHROMA was applied to the prepared surfaces as one layer. The U-Veneer template (Ultradent, South Jordan, Utah, United States) was chosen to coincide with the outline form of the tooth and was placed accordingly. The central line on the U-Veneer template was aligned with the long axis of the tooth and gently pressed [24].

Excess composite resin was removed from around the edges of the veneer to lessen the need for finishing after curing. Next, the composite resin was directly light-cured through the U-Veneer template in accordance with the manufacturer’s instructions, using the same LED curing unit. The template was then removed by pulling on the handle, leaving a highly self-polished surface. Finishing was conducted with surgical scalpel blades number 12 and 15 to eliminate any excess composite material [25].

For group II:

A layer of self-adhesive Prime&Bond was applied to the prepared enamel surfaces in the same manner as in group I. The prepared enamel surfaces were restored with Ceram.x spectra™ ST according to the previously selected shade (A1, A2, A3, A3.5, A4), placed, cured, and finished as previously mentioned for group I. The intensity of the LED curing light was checked periodically with a radiometer (Demetron Research Corp., Danburg, CT, USA) to ensure its output intensity and durability after curing every 10 veneer restorations.

- Evaluation procedures

All veneers were clinically evaluated at the beginning of the study (after 24 hours) and again at 6, 9, 12, 18, and 36 months using a modified version of the United States Public Health Service  Table 3. The parameters considered included restoration fracture, marginal adaptation, marginal discoloration, surface texture, color match, secondary caries, anatomical form, and postoperative tooth hypersensitivity. Two calibrated investigators not involved in the restoration placements conducted the clinical evaluations. The assessment was performed under a dental operating light with assistance from an intraoral camera, flat surface mouth mirrors, and a dental explorer [26]. A score of 0 represented the ideal clinical situation, a score of 1 was clinically acceptable, a score of 2 was questionable, and scores of 3 and 4 indicated unsatisfactory conditions requiring repairs or replacement of the restoration. An evaluation sheet recorded patient scores at each follow-up period.

The marginal seal clinical outcomes were verified through scanning electron microscopy (SEM) examination of inverse replicas of 32 randomly selected veneer restorations from both tested groups at different follow-up periods. A silicone impression material (Aquasil Ultra LV, Dentsply) was used to create replicas that were examined via SEM (JEOL, Jsm-6510 LV, Japan). For the complete restoration interface of the marginal veneer, these replicas were placed on custom-made aluminum stubs, gold-sputtered, and examined under a scanning electron microscope (SEM) at a magnification of 250X [27].

- Statistical analysis

Data were collected, tabulated, and statistically analyzed using the Statistical Package for the Social Sciences (SPSS) version 26 during the assessment periods. The Friedman test was used to compare the distribution of criteria scores for the same group at each assessment time, whereas the Wilcoxon signed-rank test detected significance between different evaluation periods for the same group. The Mann‒Whitney U test was used to compare the distribution of scores between the two groups of veneer restorations at each clinical evaluation period. An unpaired t test was performed to detect differences in the mean marginal gap values between the two groups at each evaluation period, and a paired t test was used to detect differences between follow-up periods within each group. A *p value* <0.05 was considered to indicate a significant difference, whereas a *p value* <0.001 was considered to indicate a highly significant difference.

## Results

**Figure 2:**
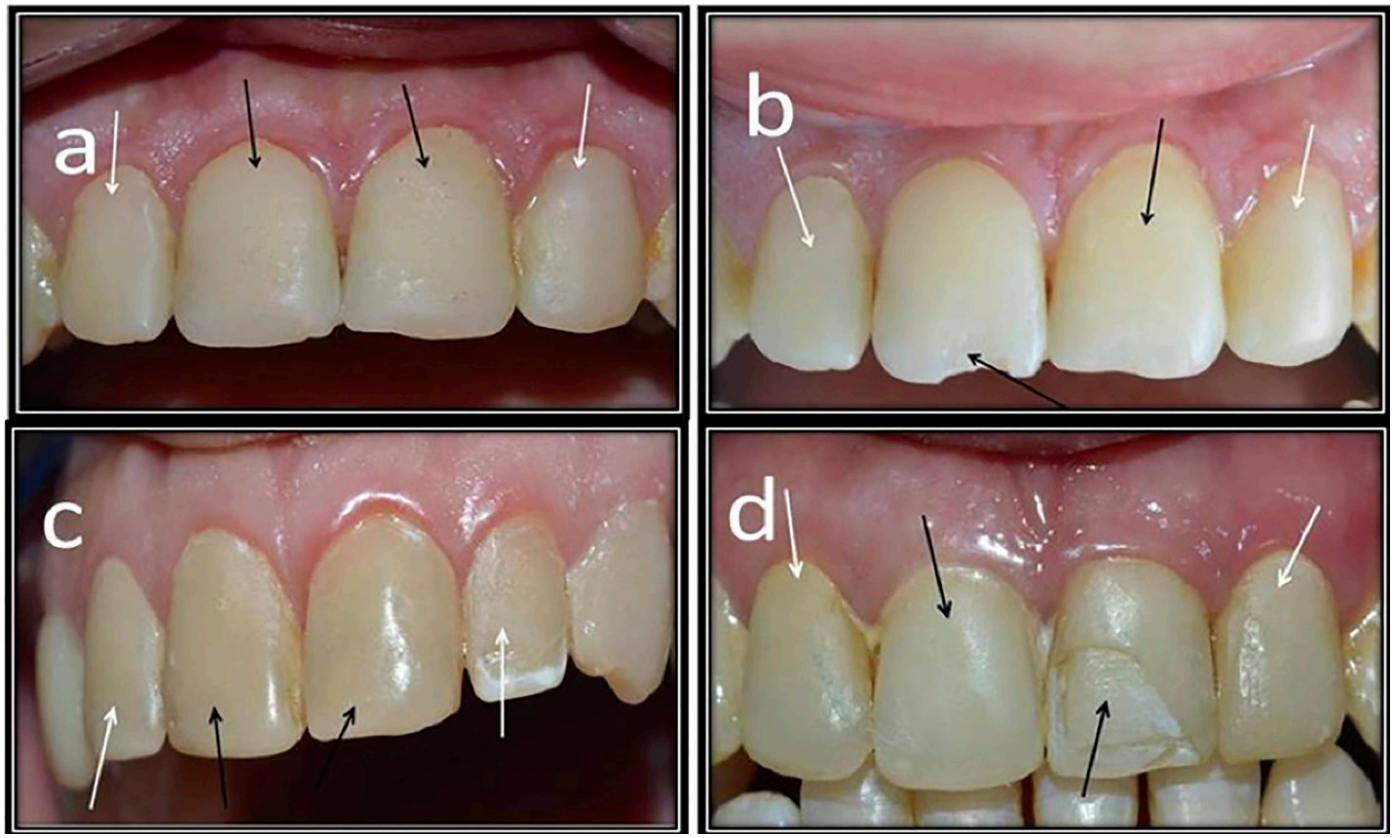
Clinical photo represents Score 0 of fracture of restoration for upper incisors at baseline. b. Clinical photo represents Score 2 of fracture of restoration for upper right central incisor veneered with OMNICHROMA at 18 months. c. Clinical photo represents Score 3 of fracture for upper left lateral incisor veneered with Ceram. x spectra™ ST at 18 months. d. Clinical photo represents Score 4 of fracture for upper left central incisor veneered with OMNICHROMA at 18 months.

The data from the clinical evaluation of all the criteria in both tested groups are presented in  Table 4. Concerning restoration fracture, only two veneer restorations (4.54%) in group I presented varying degrees of chipping (2 and 4), whereas one veneer restoration (2.27%) in group II presented a moderate degree of chipping with a score of 3 after 18 months. There were no significant differences between the distributions of fracture scores during all evaluation periods for groups I and II (*p* = 0.092 and 0.406, respectively). Additionally, there was no significant difference between the two groups, as the *p value*s were greater than 0.05. Clinical photographs are presented to clarify restoration fracture results (in Fig. 2).

**Figure 3:**
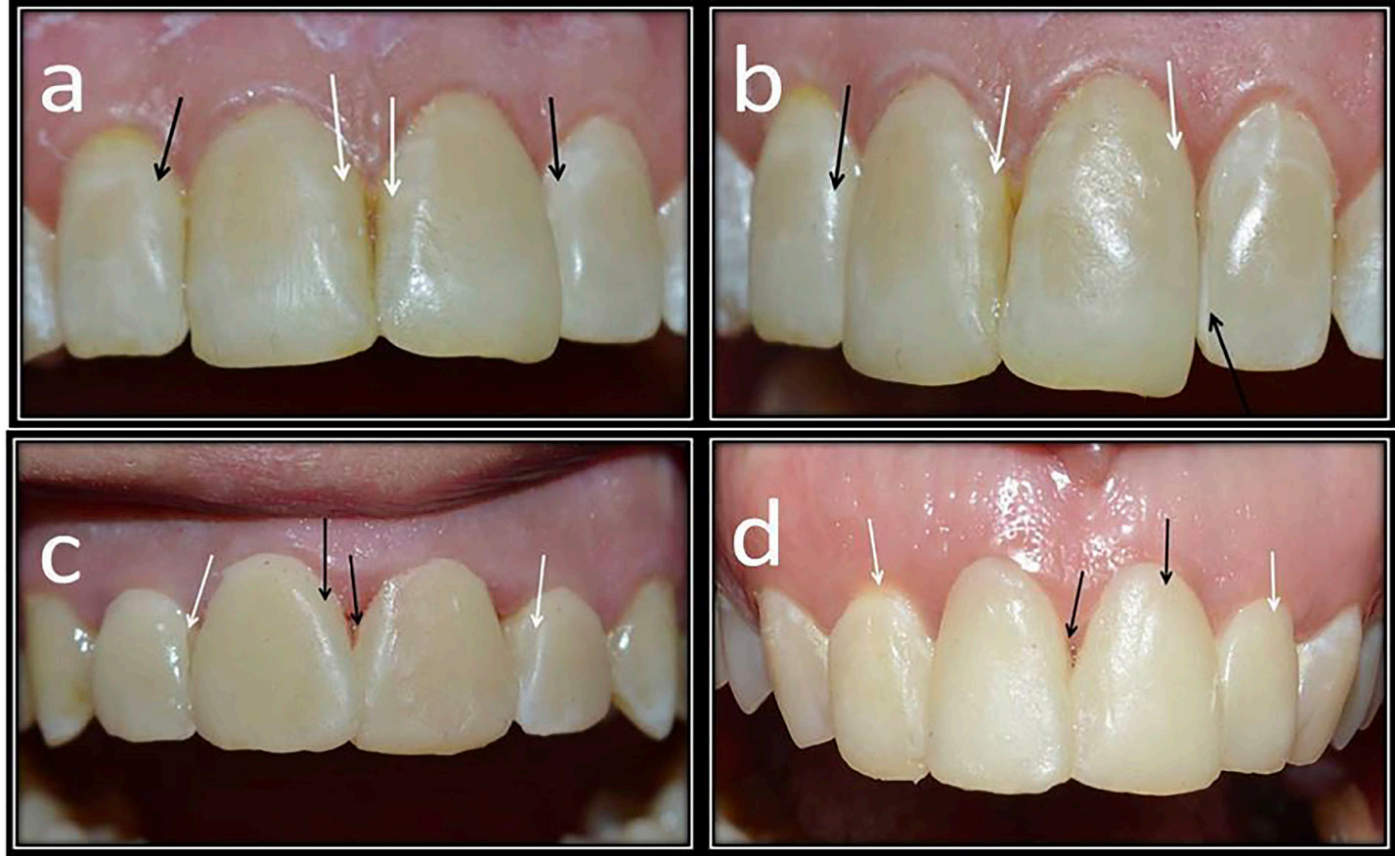
Clinical photo represents Score 0 of marginal adaptation for upper incisors at baseline. b. Clinical photo represents Score 1 of marginal adaptation for upper left lateral incisor veneered with OMNICHROMA at 18 months. c. Clinical photo represents Score 1 of marginal adaptation of upper right lateral incisor veneered with Ceram. x spectra™ at 12 months. d. Clinical photo represents Score 2 of marginal adaptation for upper right lateral incisor veneered with Ceram. x spectra™ at 36 months.

Regarding marginal adaptation, in group 1, three veneer restorations (6.82%) had a score of (1) after 12 months, four veneer restorations (9.09%) had a score of (1), one veneer (2.27%) had a score of (2), and one veneer (2.27%) had a score of (3) at 18 months. After 36 months, six veneer restorations (13.63%) had a score of (1), two veneers (4.54%) had a score of (2), and one veneer (2.27%) had a score of (3). In group II, four veneer restorations (9.09%) had a score of (1) at 12 months, whereas five veneers (9.09%) had a score of (1), and two veneers (2.27%) had a score of (3) at 18 months. After 36 months, eight veneer restorations (13.63%) had a score of (1), and two veneers (4.54%) had a score of (2). The Friedman test revealed a significant difference between groups I and II (*p* <0.05). The Wilcoxon signed-rank test demonstrated a significant difference in group I (*p value*s = 0.024) and group II (*p value*s = 0.046 and 0.014) between the results at baseline and at 6 and 9 months versus those at 18 and 36 months. The Mann‒Whitney U test revealed no significant difference between the groups, with *p value*s of 0.695 and 0.787, respectively. Clinical photographs of the marginal adaptation results are presented in Fig. 3.

**Figure 4:**
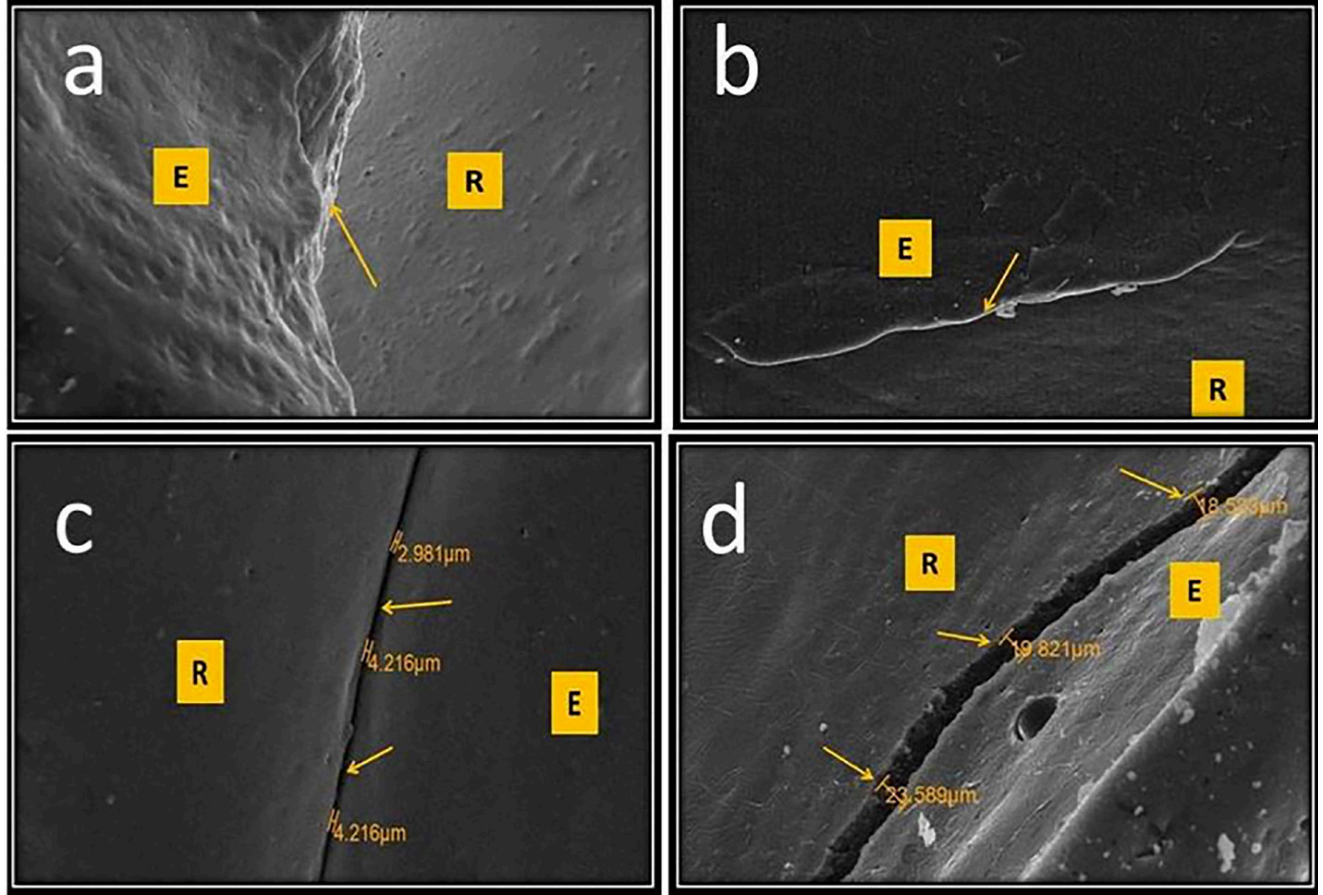
SEM image at magnification x250 of inverse replica of veneer restoration between the veneer restoration (R) & enamel tooth structure (E) a. at 9 months. b. at 12 months. c. at 18 months. d. at 36 months.

At baseline, 6 months, and 9 months, the average gap width for both groups was zero, indicating that all examined veneer restorations had a complete marginal seal. After 12 months, a mean value of 2.95 µm was recorded in group I, whereas group II had a mean gap width of 3.21 µm. After 18 months, further marginal deterioration occurred, with a mean gap width of 5.98 µm in group I and 6.80 µm in group II. After 36 months, group I presented a mean gap width of 6.85 µm, whereas group II presented a mean gap width of 7.11 µm. The Shapiro‒Wilk test was performed to assess the distribution of marginal gap width data, and the data were found to be normally distributed. The paired t test revealed a significant difference between different evaluation periods in each group (*p* = 0.000). Moreover, the unpaired t test revealed no significant difference between the two groups after the 12-, 18-, and 36-month follow-up periods (*p value*s = 0.218, 0.236, and 0.227, respectively). The results regarding the marginal seal and gap width are illustrated in Fig. 4, which shows representative SEM photomicrographs of the impression replica technique (inverse replica) of veneer restorations from both groups at various evaluation periods.

Regarding the marginal discoloration criterion, after 12 months, four veneer restorations (9.09%) had a score of (1) in group I, seven veneer restorations (15.90%) had a score of (1) at 18 months, and nine veneer restorations (20.45%) had a score of (1) at 36 months. In group II, five veneer restorations (11.36%) had a score of (1) at 12 months, six veneer restorations (13.63%) had a score of (1), and two veneer restorations (4.54%) had a score of (2) at 18 months. At 36 months, eight veneer restorations (18.18%) had a score of 1, and four veneer restorations (9.09%) had a score of 2. There was a highly significant difference between the different evaluation periods for both groups I and II, with a *p value* of 0.000. A significant difference was noted between the results at baseline, 6 months, and 9 months compared with those at 12, 18, and 36 months (*p* = 0.046, 0.008, and 0.0326, respectively), as was the case for group II (*p* = 0.025). No significant differences were recorded between the two groups, with *p value*s of 0.726, 0.711, and 0.831 after 12, 18, and 36 months, respectively.

Surface texture began to change at 12, 18, and 36 months. In group I, five veneer restorations (11.36%) had score of (1) at 12 months; in group II, this number was six (13.63%). Additionally, seven veneer restorations each in groups I and II (15.90%) had a score of (1) at 18 months, and eight from group I (18.18%) had a score of (1) at 36 months. The Friedman test revealed a highly significant difference (*p* = 0.000) between the various follow-up periods for both groups I and II. A significant difference for group I was recorded between the results at baseline, 6 months, and 9 months compared with the 12-, 18-, and 36-month follow-up periods (*p value*s = 0.025, 0.008, and 0.007, respectively), whereas for group II, the *p value*s were 0.014, 0.008, and 0.008, respectively. There was no significant difference between the two groups (*p* >0.05).

In the color match criterion analysis, the color of both tested veneer groups matched the color of the surrounding natural teeth and revealed a (100%) score of (0) after 24 hours, 6 months, and 9 months. However, the color changed over time, with eight veneer restorations (18.18%) in group I having a score of (1) at 12 months, whereas 11 veneer restorations (25%) had a score of (1) at 18 months. Furthermore, for group II, nine veneer restorations (20.45%) had a score of (1) at 12 months, while 11 veneer restorations (25%) had a score of (1), and four veneer restorations (9.09%) had a score of (2) at 18 months. At 36 months, 11 veneers had a score of (1) and three had a score of (2) in group I, whereas 12 veneers had a score of (1) and four had a score of (2) in group II. A significant difference for group I was noted between the results of the baseline, 6-month, and 9-month follow-up periods versus the 12-, 18-, and 36-month follow-up periods (*p* = 0.005, 0.001, and 0.002, respectively); these findings were also observed in group II (*p* = 0.003, 0.001, and 0.004, respectively). The Mann‒Whitney U test revealed no significant difference between the two veneer groups at any clinical evaluation time, as the *p value* was >0.05.

In the anatomical form criterion analysis, there was no change in the anatomical form except at the 18- and 36-month follow-up periods. Two veneer restorations in group I (4.54%) had scores of (1) and (2), whereas one veneer restoration (2.72%) in group II had a score of (2). The Friedman test revealed that for both group I and group II, none of the distributions of the anatomical form scores across the different evaluation periods were significant, with *p value*s of 0.092 and 0.406, respectively. The Mann‒Whitney U test revealed no significant difference between the two tested groups at any evaluation period, as indicated by *p value*s greater than 0.05. Neither tested veneer group experienced secondary caries or postoperative hypersensitivity throughout the study period.

Regarding the relationship between marginal adaptation and marginal discoloration, Spearman’s correlation test revealed a significant and strong positive relationship in group I at 12, 18, and 36 months, with correlation coefficients of rho = 0.855 (*p* <0.001), rho = 0.911 (*p* <0.001), and rho = 0.765 (*p* <0.001), respectively. The same was observed for group II, where the correlation coefficients were rho = 883 (*p* <0.001), rho = 898 (*p* <0.001), and rho = 693 (*p* <0.001), respectively. Furthermore, a strong positive and significant correlation was found between surface texture and color match for group I at 12, 18, and 36 months, as indicated by rho = 0.760 (*p* <0.001), rho = 0.753 (*p* <0.001), and rho = 0.852 (*p* <0.001), respectively. For group II, the correlations were rho = 0.784 (*p* <0.001), rho = 0.513 (*p* <0.001), and rho = 0.831 (*p* <0.001), respectively. Additionally, strong positive correlations were observed after 12, 18, and 36 months between surface texture and marginal discoloration for group I, with correlation coefficients of rho = 0.883 (*p* <0.001), rho = 1.000 (*p* <0.001), and rho = 0.961 (*p* <0.001), respectively. Similarly, for group II, the coefficients were rho = 901 (*p* <0.001), rho = 897 (*p* <0.001), and rho = 769 (*p* <0.001).

## Discussion

The field of aesthetic and restorative dentistry has rapidly evolved to provide newer and improved solutions that meet the ever-increasing demand for a more natural, lifelike appearance of dentition [28]. Clinicians should consider materials and techniques that enable the most conservative treatment while satisfying the patient’s aesthetic, structural, and biological requirements, in addition to the mechanical requirements needed to achieve clinical durability [29].

Dental veneers are widely regarded as one of the most effective treatment options for tooth discoloration, dental malformations or misalignments, enamel hypoplasia, crown fractures, and erosive or abrasive defects. Aesthetic veneers demonstrate excellent clinical performance, and as materials and techniques have advanced, veneers have become one of the most predicTable, aesthetically pleasing, and least invasive restorative treatments for enhancing patient smiles [30].

Shade selection for teeth was performed visually using the Vita Classical shade guide and through the VITA Easyshade V device. The accuracy and precision of the visual method depend on several factors, including the characteristics of the shade guide, the observer’s color matching ability or expertise, and the light source [31]. Additionally, various factors impact spectrophotometric measurements, such as the size of the surface being measured, the proper positioning and angles of the probe, and the effectiveness of the device’s color analysis software and shade guide mode. Therefore, combining both methods is highly recommended in this study [32,33].

Currently, window preparation is used as a conservative veneer preparation design that requires less tooth reduction, with a depth of 0.3–0.5 mm [34]. Silveira RC *et al*. [35] reported that window preparation design retains more of the tooth’s structure, as it does not involve reducing the incisal edge and has a lower probability of marginal discrepancy along the margins of veneer restorations than other preparation methods do.

The current research used the U-Veneer Kit to create direct composite veneers with predicTable shape and symmetry, mimicking the precise anatomic facial contour of teeth more quickly and easily. Additionally, the templates produce a highly polished glossy surface by preventing the formation of an oxygen inhibition layer during the curing process, thereby increasing the composite strength and reducing the need for finishing and polishing procedures [24,36].

The two most widely used criteria for the clinical evaluation of composite restorations are the United States Public Health Service criteria (USPHS) and the Federation Dentaire Internationale (FDI) criteria. Recent reports indicate that the FDI criteria are more sensitive than the USPHS criteria. Consequently, modified USPHS criteria were developed to increase their selectivity. The FDI criteria were applied given the trend toward their use for assessing restorations, whereas the USPHS criteria facilitated further comparisons with earlier studies [37]. Carvalho AA *et al*. [38] and Vieira RM *et al*. [39] concluded that both criteria (USPHS and FDI) were effective in the clinical evaluation process, yielding similar outcomes.

Modified USPHS criteria were used for clinical evaluation in the present study, which are frequently used for the long-term evaluation of restorations. These criteria provide a more precise, descriptive, and standardized assessment of restorations across different observation periods [40]. The USPHS criteria remain the most widely used systems in clinical trials for evaluating the important characteristics of dental veneers and are commonly regarded as valid means of generating data of significance [41].

A total of 96.59% of the veneer restorations were retained without fracture by the end of this study. This is the most reliable diagnostic criterion since it does not depend on the examiner’s subjective judgment. This result can be attributed to the high flexural strength of the tested materials. Yılmaz Atalı *P* *et al*. [42] reported that OMNICHROMA has a high flexural strength of 100–120 MPa, whereas Ceram.x spectra™ ST flexural strength values are up to 112.61 MPa, as indicated by the manufacturer. According to ISO standards, the minimum flexural strength for resin composite materials must be at least 80 MPa [43].

Both tested groups demonstrated satisfactory clinical performance in terms of marginal adaptation, with no significant differences observed. Additionally, deficiencies in marginal adaptation may stem not only from gap formation but also from an excess of the adhesive system or resin composite, which can impair proper adaptation to veneer restoration margins, regardless of the finishing procedure [44]. The impression replica technique via SEM was employed to provide both qualitative and quantitative evaluations of the veneer restoration margins [45]. The current clinical results were nearly comparable to those observed through SEM examination of the inverse replica.

Further increases in the marginal gap over time (2.95–6.80 µm) could be related to the presence of HEMA in the one-component self-etching adhesive, which adversely affects the mechanical properties and stability of the adhesive interface over time [46]. In agreement with the present results regarding the marginal gap width, Badami V *et al*. [47] conducted a study indicating that a marginal gap width of 50–120 µm is considered clinically acceptable for laminate veneers.

In agreement with the present results, Ceyda SA *et al*. [48] evaluated the marginal deterioration of two novel nanofilled resin composites and revealed that OMNICHROMA had a lower amount of marginal leakage than did 3 M Filtek Universal, with no significant difference. This finding was attributed to the increased nanofiller ratio for OMNICHROMA (79 wt%) compared with 3 M Filtek Universal (76 wt%). Additionally, Neves *P* *et al*. [49] reported that the Spectra™ ST composite exhibited less marginal deterioration than the Surefill One™ composite did because of its lower degree of polymerization shrinkage.

In contrast, Latif AR *et al*. [50] assessed the violation of the marginal fit of the material in the contact zone with the hard tissues of the tooth and noted it in 60% of the cases when the composite material Ceram.x® SphereTEC was used compared with Tetric N-Ceram and OptiShade. Additionally, Bajabaa S *et al*. [51] evaluated and compared the microleakage of different resin composites and revealed that OMNICHROMA resulted in greater marginal leakage than did the nanohybrid composites, as it has a 79 wt% filler load, which is less than that of the nanohybrid composites (Tetric N Ceram has an 80–81 wt% filler load, and Harmonize has an 81 wt% filler load).

Both tested veneer materials were clinically accepTable throughout the follow-up periods concerning the marginal discoloration criterion. This acceptance may be attributed to the presence of nanofillers, which reduce surface roughness and minimize discoloration. Additionally, the filler loading (79% by weight for OMNICHROMA and 78–80% by weight for Ceram.x spectra) decreased the likelihood of discoloration due to the degradation of uncured resin, contributing to the material’s adaptation and integrity at the tooth/resin interface [42,52].

In the present study, slight marginal staining was observed over time, potentially due to the infiltration of colored molecules into the interface and/or within the adhesive layer. The presence of water, acidic monomers, and HEMA in adhesives makes them hydrophilic, thereby increasing the water sorption of the adhesives. This process is time-driven, resulting in a more porous adhesive interface that becomes increasingly susceptible to marginal staining over time. Additionally, marginal discoloration is caused primarily by the accumulation of stains at the marginal steps, crevices, or microleakage between excess materials and uncut enamel [53].

Secondary caries were not recorded during the study period. This could be attribuTable to proper patient selection, good oral hygiene, high marginal adaptation scores, and the use of a rubber dam, which consistently resulted in a lower failure rate [54]. In agreement with the present study, Aslan YU *et al*. [55] reported that no secondary caries were observed in the laminate veneers during the evaluation periods, despite the presence of slight marginal defects and marginal discoloration. Moreover, Irgang L *et al*. [56] reported that three composite veneers experienced secondary caries after a 10-year evaluation period. This difference may be due to the variation in the evaluation periods.

The surface texture began to change at 12 and 18 months in both groups. Minor alterations in surface texture may be attribuTable to organic matrix abrasion, possibly accompanied by the appearance of bubbles enclosed within the resin composite and a loss of smaller filler particles. The results of the present study may be explained by the different attitudes of patients, who consume various types of food and use different brushing methods and toothpastes, all of which play essential roles in the clinical changes in surface roughness [57].

These results align with those of Khairy AA *et al*. [58], who investigated the changes in the surface roughness of different resin composites and revealed that the nanofill composite (OMNICHROMA) had a lower surface roughness than the nanoceramic composite (spectra™ ST). They concluded that the size of the fillers and the clustered arrangement of their particles are among the most critical factors determining the surface properties of the restoration, as nanofillers create less interparticle space, resulting in a smoother restoration surface with decreased roughness.

The results of the present study revealed that there was no change in the anatomic form of the veneer restorations except at the 18-month follow-up. The nearly comparable changes in anatomic form, with no significant difference between the two tested materials, may be attributable to the fact that all the tested composite veneers contain nanofiller sizes and technologies, providing excellent anatomic forms and high wear resistance. Additionally, the high filler content and good mechanical properties render the surface more wear resistant [59].

These findings are consistent with those of Demirci M *et al*. [60], who evaluated the medium-term clinical performance of direct composites for diastema closure and teeth recontouring via Ceram.x composites. They concluded that these composites demonstrate excellent clinical performance in terms of their anatomical form due to their highly optimized nanosized filler load, which provides outstanding mechanical properties. Additionally, Ahmed MA *et al*. [61] reported that OMNICHROMA has superior mechanical properties and long-term wear resistance compared with conventional resin composites, while also offering admirable aesthetics.

No cases of postoperative hypersensitivity were reported in either group in the present study. This finding may be because all preparations and margins of veneers were completed within the enamel of the labial surface of anterior teeth to a depth of 0.3–0.5 mm. Mature enamel is not a sensitive structure in terms of pain, as it lacks blood vessels, nerves, and cells [62]. Attia YS *et al*. [22] reported that no postoperative sensitivity was detected in minimally invasive laminate veneers because the tooth preparations were performed in enamel with superior adhesive bonding.

The color of the tested veneer restorations matched that of the surrounding natural teeth and revealed a (100%) score of 0 after 24 hours, 6 months, and 9 months. The color changed slightly over time. This study revealed clinically accepTable color match scores for both groups, with no significant difference. One possible explanation for the superior color matching abilities of the single-shade composite is that it closely reflects the shade of the original teeth due to its high translucency, as investigated by Yağcı Ö and Fidan M. [63], who concluded that the blending effect increased with greater translucency, in which the refractive index of OMNICHROMA changed from 1.47 to 1.52 after polymerization.

Gamal WM and Riad M. [64] agreed with the current results regarding the color match and blending effect of OMNICHROMA on the tooth enamel surface. This finding is explained by its reliance on innovative chromatic technology to control optical properties. It contains no pigments; thus, its color characteristics depend solely on the physical properties of light, unlike traditional resin composites, which obtain their color chemically by incorporating pigments into the material. Additionally, Korkut B *et al*. [65] conducted a two-year retrospective evaluation of OMNICHROMA monoshade direct composite veneers and reported that all OMNICHROMA restorations exhibited 100% accepTable color matching throughout the two-year period.

Additionally, the present study demonstrated clinically acceptable color match scores for Ceram.x spectra. This is explained by the advanced granulated filler technology SphereTEC™, which is patent pending. SphereTEC® fillers have been optimized to balance translucency, opacity, light absorption, and scattering, enhancing color match and aesthetic results [66]. Azami NH *et al*. [67] concluded that Ceram.x composite veneers produced remarkable aesthetic outcomes by using just five CLOUD shades of intermediate translucency, influenced by the color of the surrounding tooth structure.

These results align with those of Bilen H and Türkün SL, [68] who evaluated the clinical performance of Ceram.x direct composite veneers via U-Veneer templates over a period of 18 months and concluded that Ceram.x veneers were clinically successful in terms of color matching after the 18-month evaluation period. In contrast, Sanad M *et al*. [69] conducted an *in vitro* study to assess the color matching ability of a single-shaded resin composite (OMNICHROMA) compared with a multishade resin composite (Ceram.x). They reported that OMNICHROMA demonstrated accepTable color matching predominantly in lighter teeth shades, whereas Ceram.x exhibited superior color matching ability across three different tooth shades.

In the present research, a significant positive relationship between surface texture, marginal discoloration, and color match was observed after 12 and 18 months in groups I and II, which confirms the results obtained by Telang A *et al*. [70], who reported that the color of composite resins was affected by surface roughness and integrity. Furthermore, Chowdhury D *et al*. [71] reported that surface roughness and color change were time-dependent, as both increased over time. They explained their findings by the resin’s affinity for stains, which is modulated by its conversion rate and physicochemical characteristics, including the water sorption rate.

This study is one of the few to evaluate one-shade structurally colored resin composite veneers, and there is a lack of clinical trials reporting the use of template-assisted one-shade structurally colored and universal multishade direct veneer restorations in the literature [65]. The null hypothesis of this study was rejected because no significant differences were found in the clinical performance of the monochromatic structural colored and universal multishade direct composite veneers.

Limitations

This clinical trial has several limitations that should be acknowledged:

- Operator Blinding: Although the study was conducted using a double-blind protocol, in which both evaluators and patients were blinded, the operator could not be blinded due to the distinct handling and application protocols of the tested materials. This may have introduced a degree of operator-related bias, despite efforts to standardize all clinical procedures.

- Sample size: Although the sample size was determined through power analysis and slightly oversampled to account for potential dropouts, a larger sample size across a more diverse patient population would increase the statistical power and generalizability of the findings.

- Limited number of materials: This study evaluated only two composite materials with different shade-matching strategies (monochromatic vs. multishade). Although they were carefully selected to reflect clinically relevant options, including additional materials in future studies could broaden the scope and enhance the external validity of the results.

- Follow-up period: A 36-month follow-up period, although adequate for medium-term evaluation, may not fully capture long-term clinical behavior. Future studies with longer observation periods are necessary to thoroughly assess the durability and aging effects.

- Generalizability: The study population was relatively uniform in terms of age, oral health status, and treatment indications. Consequently, the results may not be entirely generalizable to broader or more diverse populations with complex clinical conditions.

- Single Clinical Setting: All procedures were performed in a single academic clinical setting, which may limit the external validity of the results. Multicenter clinical trials would better reflect variations in operator technique, clinical environments, and patient populations.

## Conclusions

Within the limitations of the present study, the following conclusions might be drawn:

- The clinical performance is satisfactory for both structurally colored monochromatic and universal multishade direct composite veneers.

- OMNICHROMA accelerates the restorative process and provides extensive color matching capability by removing the need for a shade assessment procedure.

- The time factor significantly impacts the assessed parameters for both composite veneer materials.

## Figures and Tables

**Table 1:** Materials used in the study.

Materials	Chemical compositions	Manufactures	Website
OMNICHROMA (Supra nano filled composite) monochromatic structural colored	Monomers: UDMA (urethane dimethacrylate), TEGDMA (tri ethylene glycol dimethacrylate); Fillers: 260 nm uniform sized supra-nano spherical silica-zirconia particles SiO2-ZrO2 (79 % by weight, 68 % by volume)	Tokuyama Dental, Tokyo, Japan	www.tokuyama -dental.com
Palfique bond (One component self-etching light-cured dental adhesive) PH (2.8)	Bis-GMA(bisphenol A-glycidyl methacrylate), TEGDMA(tri ethyleneglycol dimethacrylate),HEMA( 2-hydroxylethyl methacrylate),camphorquinone, isopropyl alcohol, purified water	Tokuyama Dental, Tokyo, Japan	www.tokuyama -dental.com
Ceram. X Spectra ST (Nanohybrid Composite) universal multi-shade A1,A2,A3,A3.5,A4	Resin matrix: poly-urethane methacrylate, triethylene glycol dimethacrylate, highly dispersed and methacrylic polysiloxane nano-particles. Filler system: blend of spherical, prepolymerized SphereTEC fillers, non-agglomerated barium glass and ytterbium fluoride. Filler load ranges from 78-80 % by weight- (60-62% by volume).	Dentsply De Trey GmbH, Konstanz, Germany	www.dentsplysirona.com
Prime&Bond Universal adhesive (universal adhesive) PH (2.5 - 3.0)	Bi- and multifunctional acrylate, Phosphoric acid modified acrylate resin Isopropanol, water, Initiator, catalysts and stabilizers	Dentsply De Trey GmbH, Konstanz, Germany	www.dentsplysirona.com

**Table 2:** Intra- oral random distribution of restorations.

Distribution of Veneer restorations	Teeth locations						Total number = 88
	Upper right central incisors	Upper right lateral incisors	Upper right canines	Upper Left central incisors	Upper left lateral incisors	Upper left canines	
Group I	10	10	2	10	10	2	44
Group II	10	10	2	10	10	2	44

**Table 3:** Modified USPHS criteria.

Category	Scores	Criteria
Fracture of restoration	0	No fracture
	1	Minor crack lines over restoration
	2	Minor chippings of restoration (1/4 of restoration)
	3	Moderate chippings of restoration (1/2 of restoration)
	4	Severe chippings (3/4 restoration)
	5	Debonding of restoration
Marginal adaptation	0	Smooth margin
	1	All margins closed or possess minor voids or defects (enamel exposed)
	2	Obvious crevice at margin, dentin or base exposed
	3	Debonded from one end
	4	Debonded from both ends
Marginal discoloration	0	No discoloration evident.
	1	Slight staining, can be polished away
	2	Obvious staining, cannot be polished away
	3	Gross staining
Surface texture	0	Smooth surface
	1	Slightly rough or pitted
	2	Rough, cannot be refinished
	3	Surface deeply pitted, irregular grooves
Color Match	0	Very good color match
	1	Good color match
	2	Slight mismatch in color or shade
	3	Obvious mismatch, outside the normal range
	4	Gross mismatch
Secondary caries	0	No evidence of caries continuous along the margin of the restoration
	1	Caries evident continuous with the margin of the restoration
Anatomical form	0	The restoration has proper anatomy and contour
	1	The restoration is slightly over contoured (bulky) or under contoured (flat). Slight deviation from normal tooth contours
	2	The restoration is decidedly over contoured (bulky) or under contoured (flat). Moderate deviation from normal tooth contours
Postoperative hypersensitivity	0	No symptoms present
	1	Slight sensitivity
	2	Moderate sensitivity
	3	Severe pain

**Table 4:** Data of clinical evaluation of all criteria in both tested groups (GI: OMNICHROMA, GII: Ceram. x spectra) at each follow-up period.

Criterion	USPHS Scores	baseline		After 6 months		After 9 months		After 12 months		After 18 months		After 36months	
		GI	GII	GI	GII	GI	GII	GI	GII	GI	GII	GI	GII
Fracture os restoration	0 1 2 3 4 5	44 0 0 0 0 0	44 0 0 0 0 0	44 0 0 0 0 0	44 0 0 0 0 0	44 0 0 0 0 0	44 0 0 0 0 0	44 0 0 0 0 0	44 0 0 0 0 0	42 0 0 0 0 0	43 0 0 0 0 0	44 0 0 0 0 0	44 0 0 0 0 0
Marginal adaption	0 1 2 3 4	44 0 0 0 0	44 0 0 0 0	44 0 0 0 0	44 0 0 0 0	44 0 0 0 0	44 0 0 0 0	41 3 0 0 0	40 4 0 0 0	38 4 1 1 0	37 5 2 0 0	35 6 2 1 0	34 8 2 0 0
Marginal discoloration	0 1 2 3	44 0 0 0	44 0 0 0	44 0 0 0	44 0 0 0	44 0 0 0	44 0 0 0	40 4 0 0	39 5 0 0	37 7 0 0	36 6 2 0	35 9 0 0	32 8 4 0
Surface texture	0 1 2 3	44 0 0 0	44 0 0 0	44 0 0 0	44 0 0 0	44 0 0 0	44 0 0 0	39 5 0 0	38 6 0 0	37 7 0 0	37 7 0 0	36 8 0 0	37 7 0 0
Color Match	0 1 2 3 4	44 0 0 0 0	44 0 0 0 0	44 0 0 0 0	44 0 0 0 0	44 0 0 0 0	44 0 0 0 0	36 8 0 0 0	35 9 0 0 0	33 11 0 0 0	29 11 4 0 0	30 11 3 0 0	28 12 4 0 0
Secondary caries	0 1	44 0	44 0	44 0	44 0	44 0	44 0	44 0	44 0	44 0	44 0	44 0	44 0
Anatomical form	0 1 2	44 0 0	44 0 0	44 0 0	44 0 0	44 0 0	44 0 0	44 0 0	44 0 0	42 1 1	43 0 1	42 1 1	43 0 1
Postoperative hypersensitivity	0 1 2 3	44 0 0 0	44 0 0 0	44 0 0 0	44 0 0 0	44 0 0 0	44 0 0 0	44 0 0 0	44 0 0 0	44 0 0 0	44 0 0 0	44 0 0 0	44 0 0 0

## Data Availability

All authors of this research paper know the research data policy. All data presented in this research paper is factual and based on precise and reliable evidence. The data supporting this study’s findings are available from the corresponding author upon reasonable request.
